# Monocytes mediate *Salmonella Typhimurium*‐induced tumor growth inhibition in a mouse melanoma model

**DOI:** 10.1002/eji.202048913

**Published:** 2021-10-29

**Authors:** Síle A. Johnson, Michael J. Ormsby, Hannah M. Wessel, Heather E. Hulme, Alberto Bravo‐Blas, Anne McIntosh, Susan Mason, Seth B. Coffelt, Stephen W.G. Tait, Allan McI. Mowat, Simon W.F. Milling, Karen Blyth, Daniel M. Wall

**Affiliations:** ^1^ Institute of Infection, Immunity, and Inflammation, College of Medical, Veterinary and Life Sciences University of Glasgow Glasgow United Kingdom; ^2^ Cancer Research UK Beatson Institute Glasgow United Kingdom; ^3^ Institute of Cancer Sciences University of Glasgow Glasgow United Kingdom

**Keywords:** Bacterial cancer therapy, Immunotherapy, Monocytes, SL7207

## Abstract

The use of bacteria as an alternative cancer therapy has been reinvestigated in recent years. SL7207: an auxotrophic *Salmonella enterica* serovar Typhimurium *aroA* mutant with immune‐stimulatory potential has proven a promising strain for this purpose. Here, we show that systemic administration of SL7207 induces melanoma tumor growth arrest in vivo, with greater survival of the SL7207‐treated group compared to control PBS‐treated mice. Administration of SL7207 is accompanied by a change in the immune phenotype of the tumor‐infiltrating cells toward pro‐inflammatory, with expression of the T_H_1 cytokines IFN‐γ, TNF‐α, and IL‐12 significantly increased. Interestingly, Ly6C^+^MHCII^+^ monocytes were recruited to the tumors following SL7207 treatment and were pro‐inflammatory. Accordingly, the abrogation of these infiltrating monocytes using clodronate liposomes prevented SL7207‐induced tumor growth inhibition. These data demonstrate a previously unappreciated role for infiltrating inflammatory monocytes underlying bacterial‐mediated tumor growth inhibition. This information highlights a possible novel role for monocytes in controlling tumor growth, contributing to our understanding of the immune responses required for successful immunotherapy of cancer.

## Graphical abstract

*Salmonella Typhimurium* can target tumors and stall their growth in mouse models, a process associated with tumor infiltration by inflammatory monocytes. Clodronate liposomes, which abrogate phagocytic cells, inhibit *Salmonella*‐mediated tumor growth inhibition, suggesting a role for these cells in *Salmonella*’s antitumor properties.

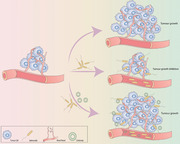

## Introduction

Since the late 19th century, bacteria and bacterial products have been studied and used clinically to treat solid tumors [1]. There is much evidence to support the antitumor effects of bacteria such as *Salmonella enterica* serovar *Typhimurium*, *Escherichia coli, Listeria monocytogenes, BCG*, and others in in‐vivo tumor models [2, 3, 4, 5, 6]. These bacteria can selectively accumulate in the tumor microenvironment and induce antitumor effects through a number of mechanisms such as metabolite depletion and direct cell killing [7, 8, 9, 10]. A primary mechanism which has been the subject of intense study is immune infiltration into the tumor following bacterial administration into tumor‐bearing mice, with many immune cells implicated in the antitumor effects which are seen to occur. Neutrophils accumulate in tumors following bacterial therapy in a number of transplantable tumor models [3], while the blockade of neutrophils in a *S*. *Typhimurium*‐treated cancer model enhanced the therapeutic effect of the bacteria by propagating the spread of the bacteria within the tumor [11]. DCs are recruited to infected tumors following *S*. *Typhimurium* administration, and those isolated from the tumor‐draining LNs produced more IL‐6, TNF‐α, and IL‐1β than DCs harvested from control mice [12, 13]. Furthermore, *S*. *Typhimurium* treatment of tumor‐bearing mice has resulted in the increased expression of connexin 43, a gap junction pore‐forming protein which facilitates the movement of tumor‐associated antigen from tumor cells to DCs [14]. Lymphocyte involvement following bacterial administration to tumor‐bearing mice has revealed somewhat contradictory findings. In one study, the depletion of CD4 using anti‐CD4 antibodies did not significantly affect the tumor‐growth inhibitory effects of *E. coli*, while the blockage of CD8 completely abolished antitumor efficacy [5]. However, previous work indicated a depletion of either CD4 or CD8 alone mildly abrogated the antitumor effects of *Salmonella enterica* serovar *Choleraesuis* [15]. Furthermore, given these reports, and others which have claimed a role for T cells in mediating the antitumor effects for bacteria, it is somewhat surprising that bacteria are also capable of tumor growth inhibition in athymic nude mice [16, 17].

Monocytes and macrophages have garnered little attention in the bacterial‐meditated cancer therapy literature. This is interesting given the critical role the cells play in mediating the immune response to oral *S*. *Typhimurium i*nfection [18, 19, 20]. These immune responses include a host of antitumor immune cells being recruited and producing an array of cytokines, such as TNF‐α and IL‐12, which are not conducive to established tumor growth [21, 22, 23]. However, it has been reported that there was an increase in the number, or density, of macrophages in the tumors following systemic bacterial administration [3, 15]. For one of these studies, the only marker used to identify macrophages was CD11b, which would also include other myeloid cells, such as DCs and neutrophils, thus, making conclusions about the role of macrophages in this setting difficult [3]. More recently, F4/80^+^ cells were credited with a pro‐inflammatory role in the tumor following infection with *S*. *Typhimurium* [6]. However, as F4/80 stains both macrophages and monocytes transitioning to macrophages, it is again difficult to determine the role the individual cell types play in the inflammatory signature and associated tumor regression.

Monocytes have yet to be thoroughly investigated for their antitumor effects in the context of cancer immunotherapy strategies. It is credible that monocytes should accumulate in the infected tumor as these cells have been shown to accumulate in inflamed tissues and provide inflammatory cytokines, such as TNF‐α, which would not be conducive to tumor progression [23, 24‐26]. The present study sought to address this dearth of information pertaining to tumor‐infiltrating monocytes following systemic administration of SL7207, an attenuated strain of *S*. *Typhimurium* [27, 28]. Here, we demonstrate that these monocytes accumulate in the tumor following systemic infection, are highly pro‐inflammatory and are potentially key immune cells required for the antitumor effects of SL7207, suggesting an important role for these cells in antitumor immune responses.

## Results

### SL7207 inhibits tumor growth and increases survival

**Figure 1:**
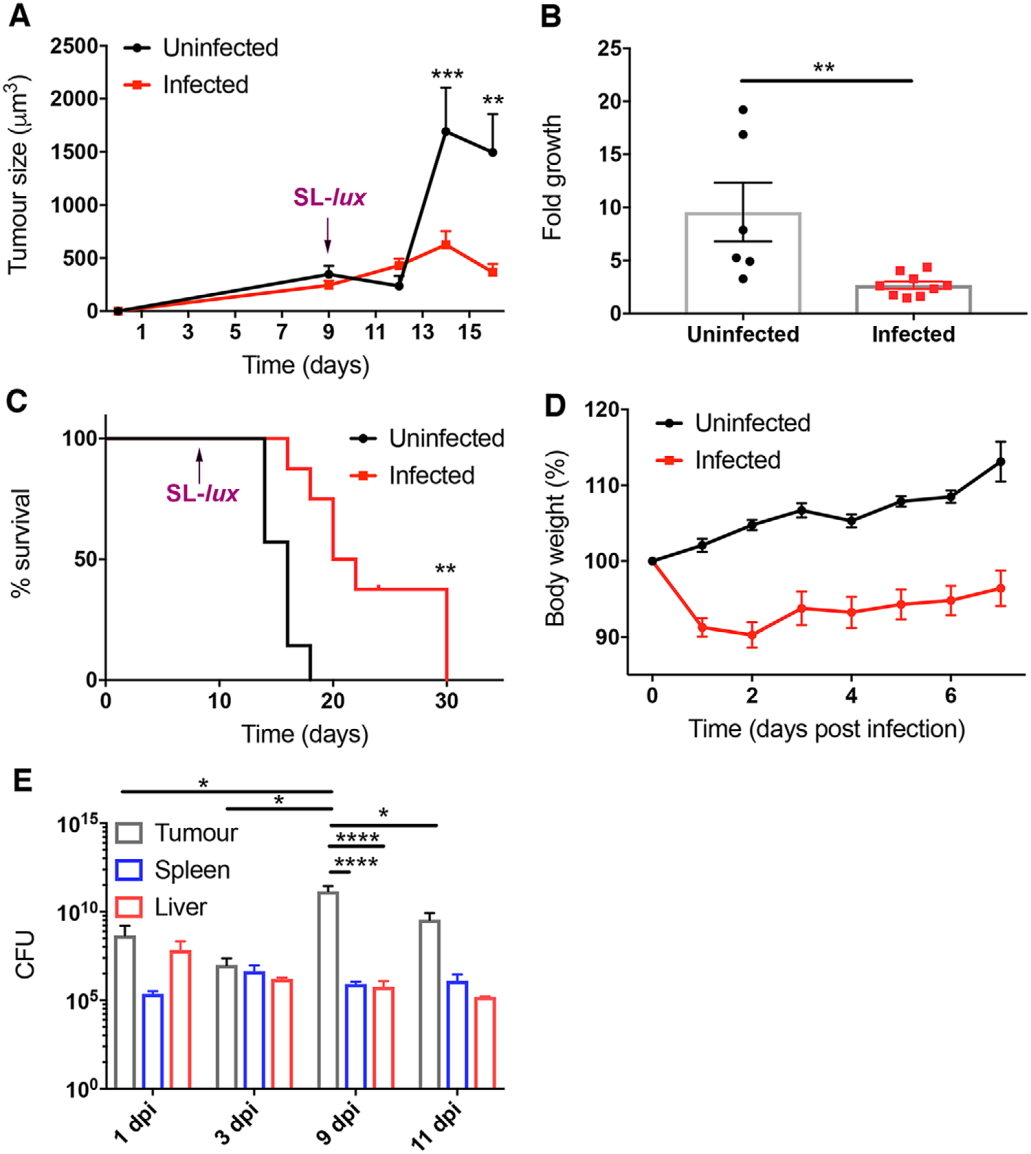
SL7207 inhibits tumor growth in B16F10 tumor models. (**A**) B16F10 tumors were allowed to develop in C57BL/6 mice. Serial tumor size measurements were taken with Vernier calipers at indicated time points (n = 4 mice). Tumor size was calculated using ^4^/_3_πr^3^ adapted from [2]. (**B**) Fold growth of tumors at the time of harvest 5 dpi compared to tumor size at the time of infection (n = at least 6 mice). (**C**) Kaplein–Meier survival curve of tumor‐bearing mice infected with SL7207 versus uninfected (purple arrows indicate time point of SL‐*lux* administration) (n = 6 mice; endpoint = 14 dpi). (**D**) Weight of mice expressed as a percentage of weight at Day 0 of infection (n = 6 mice). (**E**) Total CFUs of SL7207 were calculated at multiple time points in tumors, livers and spleens of infected mice via colony counts (n = 4 mice). Results displayed are from at least two independent experiments. Results are displayed as mean ± SD with each point representing a single animal. Samples were analyzed using a two‐way ANOVA with Sidak post‐test correction (**A**), Student's *t*‐test (**B**), Log Rank Mantel‐Cox test (**C**) or using a two‐way ANOVA with Tukey post‐test correction, (**D** and **E**) where *p < 0.05; **p < 0.01; and ***p < 0.001.

The B16F10 syngeneic melanoma model is a well‐characterized in‐vivo tumor model [29]. To validate the tumor‐growth inhibitory effects of SL7207 in this model, tumor‐bearing mice were treated with 5 × 10^6^ CFU of SL7207 in cold PBS or PBS alone via intravenous injection and tumor growth was measured for 7 days. SL7207 significantly inhibited tumor growth compared to the PBS control, and indeed the size of the tumor 7 days post SL7207 administration was not significantly increased compared to the day of infection (Figure 1A). Furthermore, there was a significantly lower fold growth of the infected tumors compared to the uninfected PBS controls from the time of treatment to the time of harvest (Figure 1B). There was also a significant improvement in survival of the infected mice compared to PBS‐treated controls at 14 day postinfection (dpi) (30 days after tumor induction; Figure 1C), however, this was also accompanied by weight loss in the infected group (Figure 1D). Finally, CFU counts of SL7207 recovered from various organs demonstrated that the total number of bacteria in the tumors increased from 1 dpi and between 3 and 9 dpi, followed by a decrease at 11 dpi (Figure 1E). There were significantly greater numbers of bacteria recovered from the tumors compared to the liver or spleen at 9 dpi.

### SL7207 administration induces a pro‐inflammatory response in the tumor

**Figure 2:**
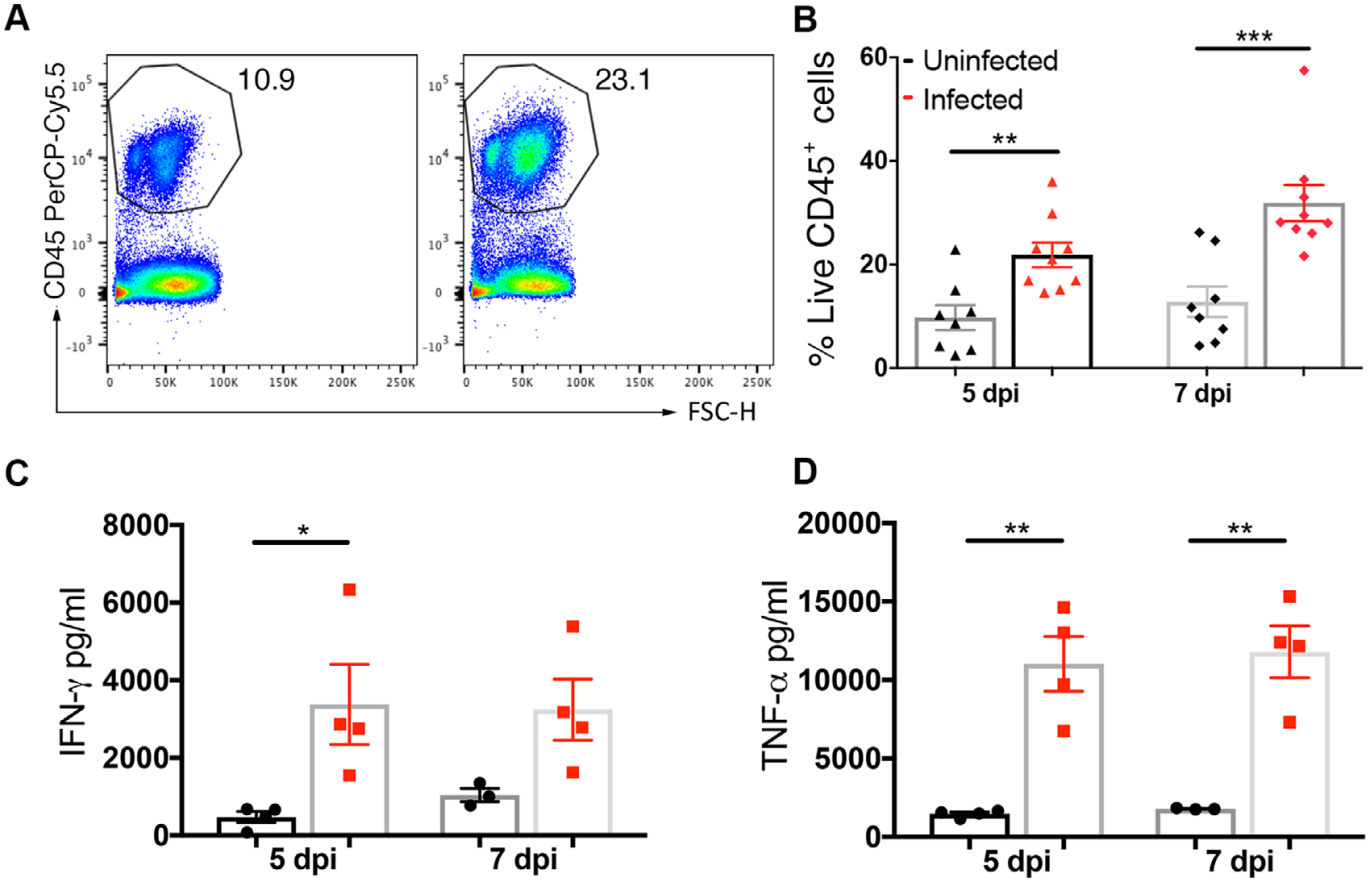
Systemic administration of SL7207 induces immune cell infiltration and pro‐inflammatory immune response in the tumor. (**A**) Representative flow cytometry plots of CD45^+^ cells in tumors. Cells were gated on live, single cells. (**B**) Percentage of live cells that are CD45^+^ (n = at least 8 mice). (**C**) ELISA analysis of tumor lysates for IFN‐γ and (**D**) TNF‐α (n = 4 mice). Results displayed (**A, C,** & **D**) are representative of two independent experiments; results displayed (**B**) are from two independent experiments. Samples for cytokine analysis were stimulated with Cell Stimulation Cocktail (eBioscience). Results are displayed as mean ± SD with each point representing a single animal. Samples were analyzed using a Student's *t*‐test where *p < 0.05; **p < 0.01, and ***p < 0.001.

To assess the immune profile of the tumor following systemic infection of SL7207, tumor‐bearing mice were infected with SL7207 or control PBS and tumors were harvested and subjected to flow cytometry or ELISA analysis. We found that there was a significant increase in the density of CD45^+^ immune cells at both 5 and 7 dpi (Figure 2A and B; see gating strategy in Supporting information Figure S1 and absolute numbers in Supporting information Figure S2). Furthermore, there was a significant increase in the amount of pro‐inflammatory cytokines IFN‐γ and TNF‐α at 5 dpi in the infected group compared to uninfected, with TNF‐α maintaining this significant increase at 7 dpi (Figure 2C and D).

### SL7207 infection is accompanied by an influx of pro‐inflammatory monocytes into the tumor

**Figure 3:**
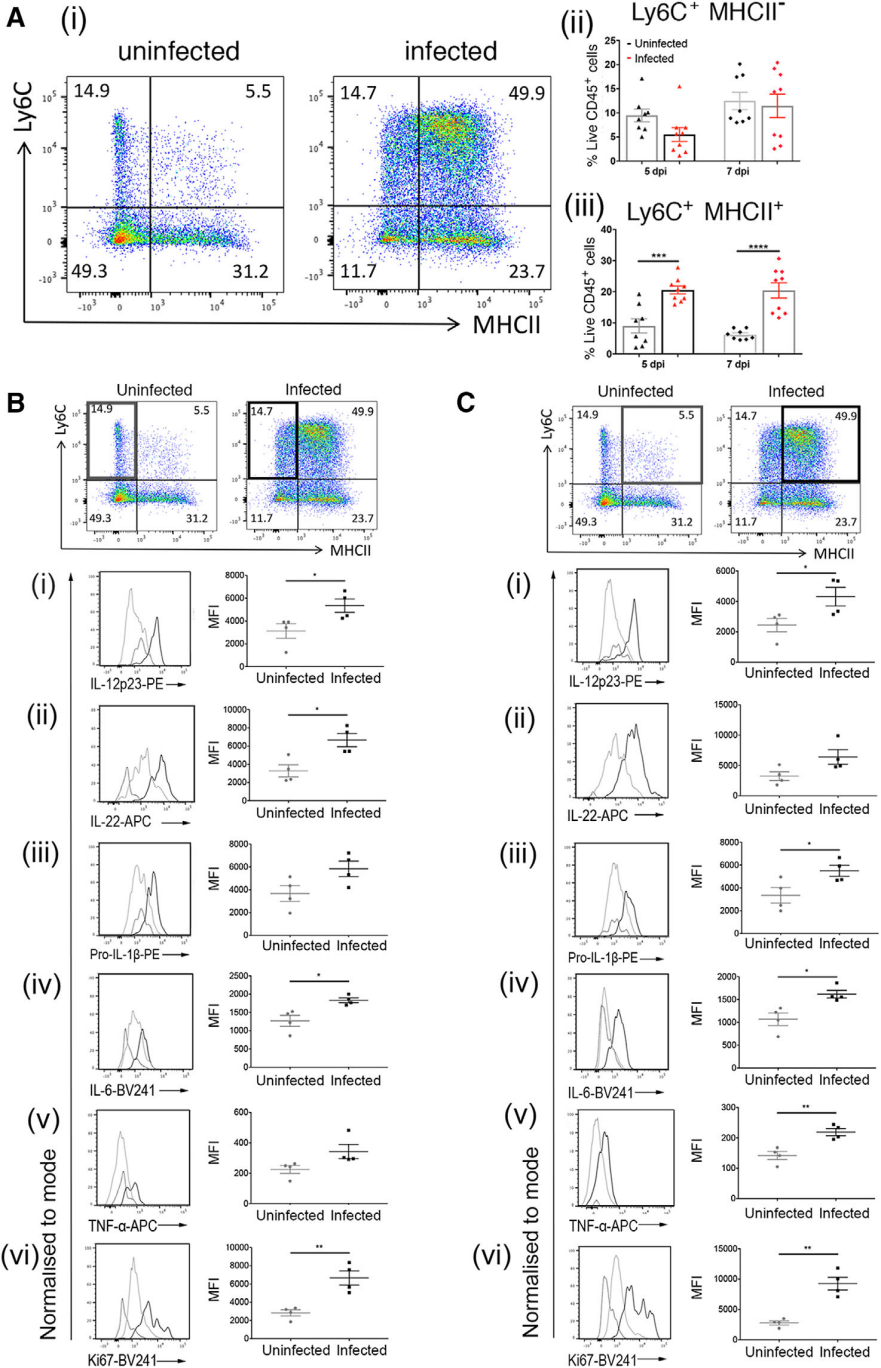
Administration of SL7207 induces Ly6C^+^MHCII^+^ monocyte accumulation accompanied by increased expression of pro‐inflammatory markers. (**A)(i)** Representative flow cytometry plots of monocyte and macrophage populations at 7 dpi. Cells gated on live, single cells, CD45**^+^**, CD11b**^+^**, SiglecF**^−^**, Ly6G**^−^**, F4/80**^+^**. **A(ii)**Percentage of CD45^+^ cells that are Ly6C**^+^** MHCII**^−^** at 7 dpi (n = at least 8 mice). **A(iii)** Percentage of CD45^+^ cells that are Ly6C**^+^** MHCII**^+^** at 7 dpi (n = at least 8 mice). (**B**) Depiction of relevant cell population (Ly6C**^+^** MHCII**^−^** monocytes, highlighted panel) with the relative expression of (**i**) IL‐12p23, (**ii**) IL‐22, (**iii**) pro‐ IL‐1β, (**iv**) IL‐6, (**v**) TNF‐α, and (**vi**) Ki67 in this population with a representative plot from each sample group: isotype control (broken grey line), uninfected (light grey line), and infected (dark grey line). (**C**) Depiction of relevant cell population (Ly6C**^+^** MHCII**^+^** monocytes, highlighted panel) with relative expression of (**i**) IL‐12p23, (**ii**) IL‐22, (**iii**) pro‐ IL‐1β, (**iv**) IL‐6, (**v**) TNF‐α, and (**vi**) Ki67 in this population with a representative plot from each sample group: isotype control (broken grey line), uninfected (light grey line), and infected (dark grey line). Representative flow cytometry plots from four mice (two from each of two independent experiments) (**A**, **B,** & **C**); all graphs (**B** & **C**) show results from two independent experiments; all plots (**Bi‐vi** & **Cu‐vi**) show quantitative data from one experiment (n = 4 mice) representative of two independent experiments. Samples for cytokine analysis were stimulated with Cell Stimulation Cocktail (eBioscience). Results are displayed as mean ± SD with each point representing a single animal. Samples were analyzed using a Student's *t*‐test where *p < 0.05; **p < 0.01; ***p < 0.001; and ****p < 0.0001.

Given the significant increase in the inflammatory status of the tumor following infection, it was pertinent to identify which immune cell types were contributing to this phenomenon. Therefore, tumors from mice infected with SL7207 or administered with PBS for 7 days were subjected to flow cytometry analysis for specific immune cell populations (Figure 3 and Supporting information Figures S3‐S6). Of particular interest was the change to the Ly6C^+^ monocyte compartment (Figure 3A(ii)). Although there was no change in the proportion of Ly6C^+^MHCII^−^ monocytes (Figure 3A(ii)), these cells were more proliferative (Ki67^+^) and secreted multiple pro‐inflammatory cytokines (Figure 3B). Ly6C^+^MHCII^+^ monocytes exhibited a significant expansion within the CD45^+^ immune cell compartment following infection (Figure 3A(iii)), and these cells also were increasingly proliferative and had increased cytokine secretion compared to PBS‐treated controls (Figure 3C). The proportion of Ly6C^−^MHCII^+^ macrophages remained unchanged between infected and uninfected samples, as did their production of pro‐inflammatory cytokines, however, there was an increase in the proliferative capacity of these cells (Ki67^+^) (Supporting information Figure S7). Finally, there appeared to be a decrease in the proportion of Ly6C^−^MHCII^−^ macrophages in the CD45^+^ population in infected samples compared to uninfected controls (Supporting information Figure S7). However, there were no differences in the production of pro‐inflammatory cytokines between the two groups of mature macrophages, although the infected samples exhibited a greater proliferative capacity compared to uninfected controls (Supporting information Figure S7).

### Administration of clodronate interferes with the tumor‐growth inhibitory effects of SL7207

**Figure 4:**
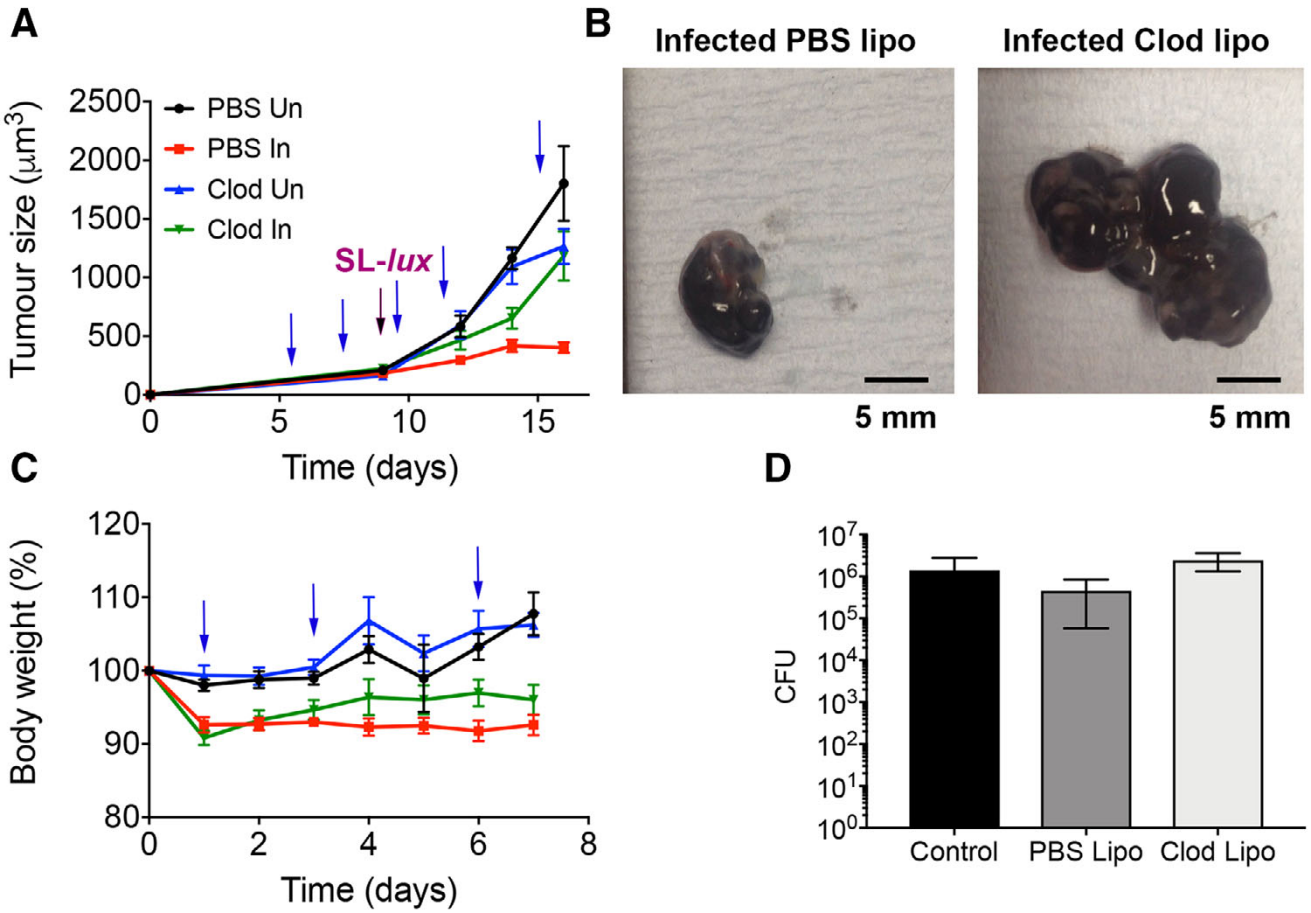
Clodronate liposome (Clod lipo) administration protects tumors from SL7207‐mediated growth inhibition. (**A**) B16F10 melanoma tumors were allowed to develop in C57BL/6 mice (n = at least 3 mice) with serial measurements and were subjected to PBS Lipo administration or Clod Lipo administration (blue arrows) with or without SL7207 infection (purple arrow). (Un = uninfected, In = infected) (**B**) Representative photographs of tumors from infected PBS Lipo and Clod Lipo tumors at 7 dpi (scale bar 5 mm). (**C**) Weight of mice in different groups (refer to Figure 4A) expressed as a percentage of weight at Day 0 of infection/PBS administration following treatment with PBS Lipo or Clod Lipo (blue arrows) (n = at least 3 mice). (**D**) CFU of tumors for indicated conditions at the time of harvest with “control” referring to no liposome treatment (7 dpi) (n = 3 mice). Results displayed (**A** & **C**) are from two independent experiments; results displayed (**D**) are from one experiment representative of two independent experiments. Results are displayed as mean ± SD. Samples were analyzed using two‐way ANOVA with Sidak post‐test correction (**A**), two‐way ANOVA with Tukey post‐test correction, (**C**) or a one‐way ANOVA (**D**) where *p < 0.05.

Given the increase in production of IL‐1β, TNF‐α, IL‐12, IL‐22, and IL‐6 by the expanded inflammatory Ly6C^+^MHCII^+^ monocyte population in the tumor following the administration of SL7207, it was hypothesized that monocyte populations may underpin the tumor‐growth inhibition effects of SL7207. Therefore, clodronate liposome treatment which depletes phagocytic cell populations in vivo (Supporting information Figure S8), was employed. Tumor‐bearing mice were treated with clodronate liposomes or PBS liposomes at multiple time points over the course of the experiment (Figure 4A) in line with previously published protocols [30, 31]. Mice were also either infected with SL7207 or treated with control PBS, and tumor growth was monitored for 7 days before tumors were harvested for analysis. Tumor‐bearing mice that were treated with PBS liposomes and subsequently infected with SL7207 showed significantly reduced tumor growth compared to uninfected PBS control mice at 7 dpi (*p* < 0.0001). However, in the clodronate liposome‐treated groups, the infected tumors grew similarly to the uninfected, PBS control tumors (*p* = 0.7929). Furthermore, the tumors from the clodronate liposome‐treated infected mice were significantly larger in size than the PBS liposome‐treated infected tumors (Figure 4A and B; *p* = 0.0001). The clodronate liposome‐treated infected mice did not have the same degree of weight loss as the PBS liposome‐treated infected mice (Figure 4C). Thus, it appears that treatment with clodronate liposomes abrogated the antitumor effect mediated by SL7207 treatment. To rule out the possibility that the liposome treatment had an effect on bacterial colonization of the tumors in infected mice, tumors were collected from all groups at 7 dpi and bacterial CFUs were compared. There were no significant differences in the CFU counts between experimental groups in any of the organs at this time point (Figure 4D). Harvesting of tumors at 7 dpi indicated the dynamic nature of immune cell infiltration into the tumor. Despite depletion of monocytes by clodronate in uninfected tumors, in contrast to, a lack of depletion seen in other phagocytic cells such as neutrophils and DCs (Supporting information Figure S8), monocyte numbers were not significantly depleted in infected tumors harvested at 7 dpi (Supporting information Figure S9). We hypothesized that infection resulted in persistent infiltration of monocytes with levels at 7 dpi did not significantly decreased, possibly due to the last clodronate treatment 2 days prior to harvesting. However, we confirmed that no other phagocytic cells were significantly depleted by clodronate treatment in infected tumors (Supporting information Figure S10,S11).

### Clodronate liposomes treatment decreases pro‐inflammatory status of tumor postinfection

As it was hypothesized that the pro‐inflammatory monocytes were mediating the antitumor effects of SL7207, it was pertinent to investigate the inflammatory status of the tumor following liposomal treatments with and without infection. This was achieved by harvesting the tumors at 7 dpi and measuring the cytokines, IFN‐γ, TNF‐α, and IL‐12 by ELISA in supernatants stimulated with Cell Stimulation Cocktail (eBioscience). While the data are representative of a single experiment, there was a significant increase in the production of all three cytokines by the PBS liposome‐treated, infected samples compared to the uninfected controls (Supporting information Figure S12). However, this increase in cytokine levels in infected samples was hindered for the clodronate liposome and infected samples compared to uninfected controls. This suggests that clodronate treatment decreases the pro‐inflammatory status of the tumors following infection.

## Discussion

The ability of bacteria, in particular *Salmonella enterica*, to induce tumor growth inhibition is well documented in the literature [2, 12, 15, 25,]. However, there remain numerous questions as to the mechanisms underlying this effect. Some studies have focused on metabolic factors, others on migratory factors but also many on the immune response mediated following infection. However, in those that examine the immune response, roles have been suggested for neutrophils, DCs, and T‐cell subtypes in contributing to the tumor‐growth inhibitory effects of bacteria [5, 11, 12, 25]. Here, we focused on the contribution of the monocyte compartment to the antitumor effects of the attenuated *Salmonella enterica* serovar Typhimurium strain SL7207, a strain known to slow tumor growth in vivo.

While it has previously been demonstrated that SL7207 has an antitumor effect in multiple in vivo models [2, 5], it was pertinent to validate the effects of SL7207 in our model. The melanoma model employed in this study is based on the use of metastatic melanoma cells, thus, these tumors tend to grow more quickly than other models, restricting the time frame of the experiments presented herein to approximately 16 days from the initial tumor cell administration to the mice. Initial work showed that the model was sufficient to investigate the antitumor and prosurvival effects of SL7207, as well as weight loss and infection in other organs (Figure 1).

The tumor microenvironment is one of an immunosuppressive phenotype, which limits the ability of the immune system to reject the tumor [32]. Tumors are often host to a high density of T_reg_ which arrest antitumor immune responses using inhibitory receptors, such as PD‐1 and CTLA‐4, resulting in the inhibition of APCs [33, 34]. Furthermore, these cells and others produce immunomodulatory molecules such as IL‐10, IL‐4, and TGF‐β [35, 36, 37]. In the present study, it is demonstrated that the administration of SL7207 is accompanied by an influx of immune cells into the tumor environment. Furthermore, these immune cells contribute to cytokines, such as TNF‐α  [20], which is known to have antitumor effects in vivo [23]. These findings are consistent with human studies. In one of these, in patients with colorectal cancer, patients with high T_H_1 cytokine production, such as IFN‐γ and TNF‐α, had prolonged survival [38].

It has been demonstrated that in up to 80% of clinical studies of cancer, increased macrophage density correlated with poor prognosis [39]. Tumor‐associated macrophages are implicated in tumor progression in multiple ways. Tumor‐associated macrophages can produce immunomodulatory cytokines [40, 41], express immunoinhibitory molecules [42, 43, 44], stimulate tumor neovascularization [45, 46], and suppress T‐cell function [32, 47]. It has been shown that tumor‐associated macrophages derived from circulating CCR2^+^ bone‐derived monocytes [48]. Both monocytes and macrophages are activated following inflammation and bacterial infection [20, 24]. The acquisition of Ly6C^hi^ monocytes is increased following inflammation in the colon [24, 26] and these cells, which normally give rise to tissue macrophages, halt in their differentiation pattern, and remain as Ly6C^+^MHCII^+^ “intermediate” cells. Furthermore, these cells appear to have greater TNF‐α production than mature macrophages and are more responsive to toll‐like receptor stimulation, suggesting that they are prime contributors to inflammation [24]. This phenomenon is also seen in the present study, whereby there is an accumulation of Ly6C^+^MHCII^+^ monocytes in the tumors following infection. These cells appear to be the principal contributors to the production of inflammatory cytokines in the monocyte/macrophage compartment following infection as neither of the tumor‐associated macrophage populations, MHCII^−^ nor MHCII^+^, produced greater quantities of pro‐inflammatory markers with infection compared to PBS controls. This observation suggests that the mature macrophages in the tumor are not capable of switching their phenotype toward a T_H_1‐cytokine‐producing state as suggested in previous publications [49]. Taken together, these data suggest that the inflammatory state demonstrated in the tumor following infection is most likely due to the accumulation of monocytes that are recruited following infection. To our knowledge, the present study is the first to describe this phenomenon in tumor inflammation. Other studies on bacterial‐mediated cancer treatment have suggested a role for macrophages in mediating antitumor effects [3, 6]. The data provided here indicate that recently recruited monocytes, not more mature macrophages as was previously suggested, were the more significant contributors to creating a pro‐inflammatory tumour microenvironment following treatment with SL7207. In the present study, the requirement of monocytes was investigated with clodronate liposomes, which target and deplete phagocytic cells. The administration of clodronate liposomes abrogated the tumor growth‐inhibitory effects of SL7207 highlighting the necessity of phagocytic cells to mediate the antitumor effects of the bacteria. We concluded that monocytes were the cell type responsible, as monocytes were the only immune cells increased at postinfection of the tumor and were the only phagocytic type depleted by clodronate within tumors. Additionally, monocytes from the tumor were pro‐inflammatory and inflammation was reduced significantly by clodronate treatment. While the expected decrease in monocyte numbers postclodronate treatment was not detected in infected tumors at day 7 postinfection, we hypothesized that clodronate depletion of monocytes was undermined by the continuous increase in bacterial numbers from day 3 of infection resulting in continued monocyte infiltration. A similar phenomenon has been observed in the intestine of C57BL/6 mice postinfection with *S. Typhimurium* with the highest, and continually increasing, infiltration of inflammatory monocytes occurring at the latest times postinfection. It is highly likely a similar phenomenon occurs here in response to increased intratumoral bacterial burden at later time points. This would result in clodronate treatment being effective early on in depleting the lower number of monocytes, in a similar manner demonstrated in uninfected tumors, before becoming increasingly overwhelmed as infection progresses. Therefore, to our knowledge, this is the first study to demonstrate that the effects of *S*. *Typhimurium* infection on tumors can be blocked by using clodronate to target phagocytic immune cells. Our data implicate monocytes in this phenomenon based on their depletion by clodronate and their important role in infected tumors post‐*S*. *Typhimurium* infection. Generalization of these findings into other tumor models and mouse strains is now needed to understand the wider significance of these cells in combatting tumors.

These findings are of therapeutic interest for cancer treatment. First, they reiterate the important contribution of monocytes and macrophages to the tumor microenvironment, particularly, the impact these cells can have on making this environment antitumorigenic when they are suitably stimulated. Second, it suggests that these cells may be manipulated to slow tumor growth. This could be achieved by bacteria, as has been shown here, or perhaps also with other monocyte‐activating agents. Finally, this study contributes to our understanding of the immune responses required to mediate effective cancer immunotherapy, providing an opportunity for further investigation into these cells in future studies.

## Materials and methods

### Bacterial strains

*Salmonella enterica* serovar Typhimurium SL7207 (Δ*aroA*, Δ*hisG*) was kindly provided by Prof. Siegfried Weiss Helmholtz Centre for Infection Research) [27, 28]. Bacterial cultures were maintained on LB agar supplemented with appropriate antibiotics. Luciferase expressing (*lux*) SL7207 (SL‐*lux*) was generated using the method of Ref. [50].

### Cell lines and animals

The B16F10 mouse melanoma cell line was kindly provided by Prof. Gerry Graham (University of Glasgow) and cells were maintained in DMEM (Gibco^®^, 12491) supplemented with 10% fetal calf serum (FCS), 1 mM l‐glutamine, 2 mM sodium pyruvate, and 100 IU/mL penicillin/streptomycin at 37**°**C and 5% CO_2_. All cells were routinely tested for mycoplasma contamination. Five‐ to eight‐week‐old female C57BL/6 mice were purchased from Charles River Laboratories. All animal procedures were approved by internal University of Glasgow and Beatson Institute ethics committee and were carried out in accordance with the relevant guidelines and regulations as outlined by the UK Home Office (PPL70/8584 and PPL70/8645).

### Infection of tumor‐bearing mice and recovery of bacteria from tissues

Six‐ to nine‐week‐old female C57BL/6 mice were inoculated subcutaneously in the right back flank with 2 × 10^5^ B16F10 cells in cold, sterile PBS. Mice‐bearing melanoma tumors of more than 200 μm^3^ (8–9 days post‐tumor cell transfer) were i.v. injected with 5 × 10^6^ CFU SL7207‐*lux* in 100 μL or 100 μL sterile PBS as a control. Tumor growth was measured throughout with Vernier calipers. For the recovery of bacteria, melanoma tumor, spleen, and liver were carefully resected, weighed, and placed in ice‐cold PBS. Tissues were then placed in 1–2 mL of ice‐cold PBS in 5 mL bijoux and homogenized using a hand held tissue homogenizer (OMNI International Inc., TM125‐220). Homogenates were then serially diluted in PBS and these dilutions were plated out on Lysogeny broth agar. Plates were checked for bioluminescent light emission to ensure SL7207‐*lux* isolation using the in vivo imaging system (IVIS; Perkin Elmer).

### Flow cytometry

Tissues were carefully resected, weighed, and placed in ice‐cold PBS. Tumors were then transferred to a digestion medium composed of 3 mg/mL collagenase A (Sigma, 10103586001) and 25 μg/mL DNAse I (Sigma, 10104159001) in DMEM [51]. All tissues were digested at 37**°**C for 30 min with intermittent vigorous shaking before being passed through a 70 μm strainer (VWR, 734‐0003) and neutralized with 8% FCS‐DMEM buffer. Tumor cells were treated with 1 mL RBC lysis buffer (Sigma, 11814389001) for 5 min at room temperature, neutralized with 10 mL of 8% FCS‐DMEM, and were centrifuged at 400 − *g* for 5 min at 4°C. Cells were resuspended in flow cytometry buffer (FB: 2% FCS, 3 nM EDTA (Sigma, E9884) in PBS). A portion of the cells was counted using Trypan Blue exclusion dye (Sigma, T8154). Single cell suspensions were washed and resuspended in FB. Cells were first stained in Fixable Viability Dye eFluor^®^ 780 (eBioscience) in PBS for 15–20 min on ice, in the dark. Following this incubation, cells were washed in 5 mL of FB and centrifuged at 400 × *g* for 5 min at 4°C. Cells were resuspended in their residual buffer before being incubated with anti‐CD16/CD32 (“Fc Block”) to reduce nonspecific binding to Fc receptors (Biolegend). After 5 min, 100 μL of extracellular antibody mixes (final dilution of 1:200 for each) were added to the cells and left on ice for 20 min before being washed with PBS and pelleted as before. Antibodies used were against: CD11b (Clone M1/70, Biolegend), CD11c (clone N4/18, Biolegend), F4/80 (clone BM8, Biolegend), Ly6G (clone 1A8, Biolegend), MHCII (clone M5/114.15.2, Biolegend), CD45 (clone 30‐F11, Biolegend), Ly6C (clone HK1.4, eBioscience), and SiglecF (clone E50‐2440, BD Biosciences). Cells were analyzed using the FACS AriaIII, LSRII analyzer, or Fortessa analyzer (all BD Biosciences) according to recommended guidelines [52]. All data generated were analyzed using FlowJo software (Tree Star Inc., Oregon, USA).

### Intracellular cytokine analysis

Single cell suspensions were washed and resuspended in FB. For intracellular cytokine analysis, cells were resuspended in 500 μL of eBioscience^TM^ Cell Stimulation Cocktail (plus protein transport inhibitors) in RPMI medium supplemented with 10% FCS, 1% l‐glutamine, and 0.01% β‐mercaptoethanol and the technique was carried out as previously outlined [53]. These cells remained at 37°C, 5% CO_2_ for 4 h before being washed in FB, centrifuged at 400 × *g* for 5 min at 4°C and subjected to surface staining as above. Following overnight incubation in Fix/Perm buffer, cells were washed twice with 2 mL Perm Buffer (both Foxp3/Transcription Factor staining buffer set, eBioscience). Cells were then resuspended in 200 μL of Perm Buffer supplemented with intracellular antibodies (1:200 for each) for 1 h in the dark. Antibodies used were against: Ki67 (clone 16A8, Biolegend), IFN‐γ (clone 554413, BD Biosciences), IL‐6 (clone MP520F3, BD Biosciences), TNF‐α (clone MP6‐XT22, Biolegend), IL‐12p23 (clone C15.6, Biolegend), pro‐IL‐1β (clone NJTEN‐3, eBioscience). Appropriate isotype controls for intracellular stains were included in all experiments.

### ELISA

For the sandwich ELISA protocol, supernatants were harvested from tumor cell suspensions which were stimulated in vitro with eBioscience^TM^ Cell Stimulation Cocktail in RPMI medium supplemented with 10% FCS, 1% l‐glutamine, and 0.01% β‐mercaptoethanol for 4 h. Supernatants were normalized to protein concentration before being subject to an ELISA protocol or TNF‐α (Biolegend), IL‐12 (Biolegend), or IFN‐γ (Biolegend), according to the manufacturer's instructions. Briefly, ELISA plates (Nunc^TM^ Maxisorp^TM^) were coated overnight with 100 μL of the relevant capture antibody at 4°C. Plates were washed three times with 0.05% PBS‐Tween (PBS‐T) and incubated with 200 μL of Blocking Solution for 1 h at room temperature before being washed three times. Samples were then added to the plates, 100 μL of each in duplicate, as well as the diluted standards. Plates were incubated for 2 h at room temperature before being washed three times with PBS‐T. Following these washes, avidin‐HRP conjugate in blocking buffer (1:500 dilution) was added to each well and incubated at room temperature for 30 min, before plates were stringently washed five times with PBS‐T. TMB Substrate Reagent (1:1 mixture of Reagent A and Reagent B) was added to each well in 100 μL for up to 60 min for color development. The OD of the plates was read at 450 nm on a FLUOstar OPTIMA Microplate reader (BMG Labtech).

### Clodronate depletion

C57BL/6 mice were inoculated with B16F10 tumor cells as described above. Four days prior to infection, mice were administered 100 μL of Clodronate Liposomes (Liposoma) via tail vein injection [30, 31, 54]. This corresponds to an approximate concentration of 0.5 mg/20 g mouse weight. At 1 day prior to infection and 1, 3, and 5 dpi, mice received 200 μL Clod Lipo. At these time points, control PBS Lipo was also administered to control PBS Lipo mice. At 7 dpi, tissues were harvested and processed as described.

### Statistical analyses

Values are represented as means and standard deviations. All statistical tests were performed with GraphPad Prism software, version 7.0c. Specific statistical tests and replicates are indicated in figure legends. Values were considered statistically significant when *p*‐values were *p < 0.05; **p < 0.01, ***p < 0.001; ****p < 0.0001.

## Author Contributions

S.A.J. was awarded the funding, designed, and performed the experiments, analyzed the data, and prepared the manuscript. M.J.O. assisted in experimental design, animal experiments, and ELISA. H.M.W. assisted in experimental design and animal experiments. H.E.H. and A.B.B. assisted with animal experiments. A.M. provided technical assistance throughout. S.M. assisted with animal experiments and provided training. S.B.C., S.W.G.T., A.M.M., and S.W.F.M. assisted with experimental design and data analysis and provided reagents. K.B. was awarded the funding, assisted in experimental design, analyzed the data, and provided reagents. D.M.W. was awarded the funding, developed the initial concept, designed experiments, and prepared the manuscript. All authors contributed in editing the manuscript for publication.

## Conflict of interest

The authors declare no commercial or financial conflict of interest.

### Peer review

The peer review history for this article is available at https://publons.com/publon/10.1002/eji.202048913

**Abbreviations**
dpi: day postinfection
FCS: fetal calf serum

## Supporting information



Supporting Information

## Data Availability

The data that support the findings of this study are available from the corresponding author upon reasonable request.
