# Supplementary material for: Monocytes mediate Salmonella Typhimurium‐induced tumor growth inhibition in a mouse melanoma model
Source: Eur J Immunol. 2021 Oct 29;51(12):3228–38. doi: 10.1002/eji.202048913 (PMC9214623; doi:10.1002/eji.202048913)
Supplement: Supplementary file 1 — Supporting Information [file EJI-51-3228-s001.docx]

**Supplementary Figures**

**Figure S1**


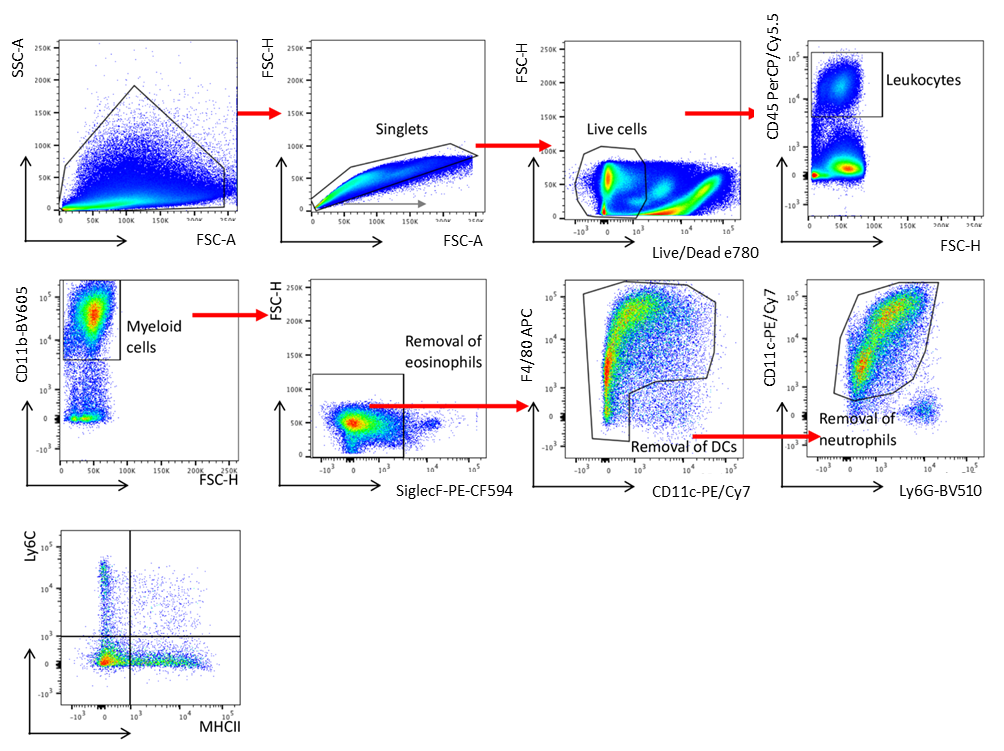


95.5

98.8

39.5

10.5

82.0

96.1

93.7

93.1

14.9

5.5

49.3

31.2

**Figure S1** **Gating strategy for the monocyte/macrophage compartment.** Cells were gated as single cells (side scatter area (SSC-A) versus forward scatter area (FSC-A)), single (FSC-A v forward scatter height (FSC-H)), live, CD45^+^, CD11b^+^, SiglecF^-^ (to exclude eosinophils), CD11c^-^ (to remove dendritic cells), F4/80^+^, Ly6G^-^ (to exclude neutrophils) and thereafter designated according to expression of Ly6C^+^ (monocytes) or Ly6C^-^ (macrophages) and on MHCII expression.

**Figure S2**


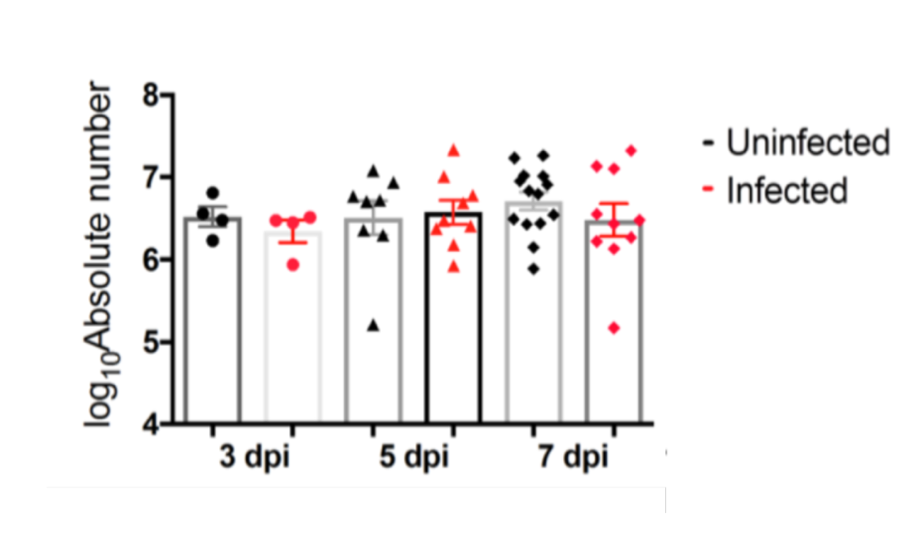


**Figure S2 Effect of SL7207 infection on absolute numbers of tumour CD45^+^ cells.**

Tumour-bearing mice were inoculated with SL7207 or PBS and tumours were harvested at 3, 5 and 7 dpi for flow cytometry analysis of tumour immune cell content. Cells were gated on single, live. Quantification of absolute number of CD45^+^ immune cells from infected and uninfected tumours at the indicated time points. Results displayed are from at least two independent experiments. Results are displayed as the mean ± SEM with each point representing a single animal. Statistical analyses performed using Students t test between infected and uninfected samples at the same time point.

**Figure S3**

**
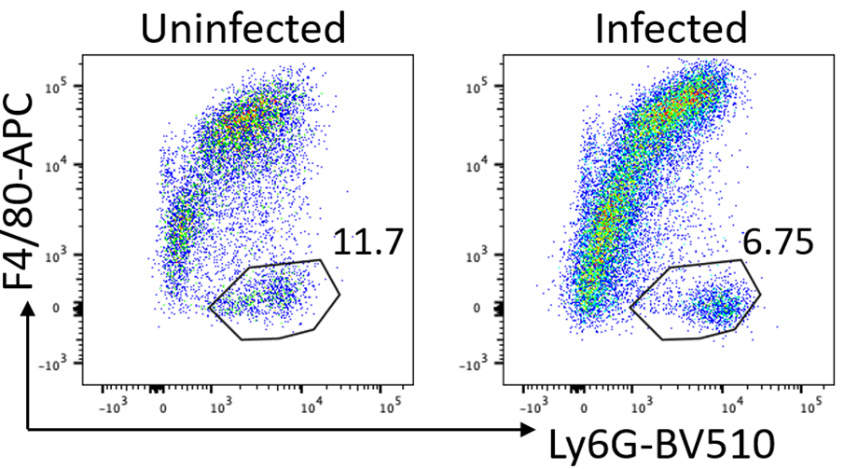
**

**A**


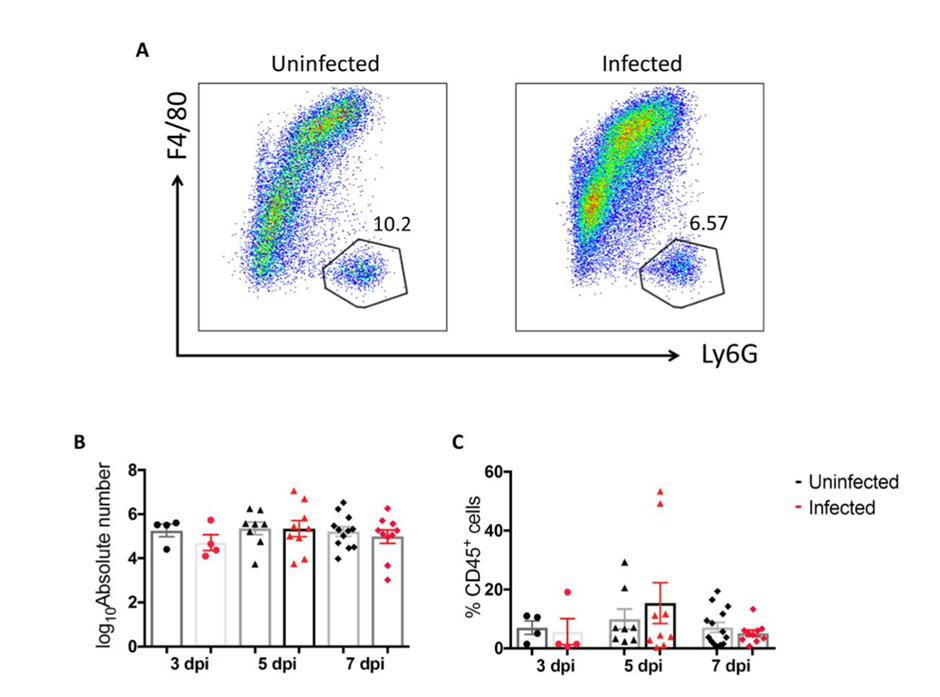


**Figure S3 Effects of SL7207 on tumour neutrophil content.** Tumour-bearing mice were inoculated with SL7207 or PBS and tumours were harvested at 3, 5 and 7 dpi for flow cytometry analysis of tumour immune cell content. **A.** Representative flow cytometry plots of Ly6G+ neutrophils from infected and uninfected tumours at 5 dpi. Cells were gated on single, live, CD45+, F4/80-. **B.** Quantification of absolute number of neutrophils from infected and uninfected tumours at the indicated time points. **C.** Data shown as percentage Ly6G+ neutrophils of total CD45+ cells from infected and uninfected tumours at the indicated time points. Results displayed are from at least two independent experiments. Results are displayed as the mean ± SEM with each point representing a single animal. Statistical analyses performed using Students t test between infected and uninfected samples at the same time point where p < 0.05*.

**Figure S4**

**
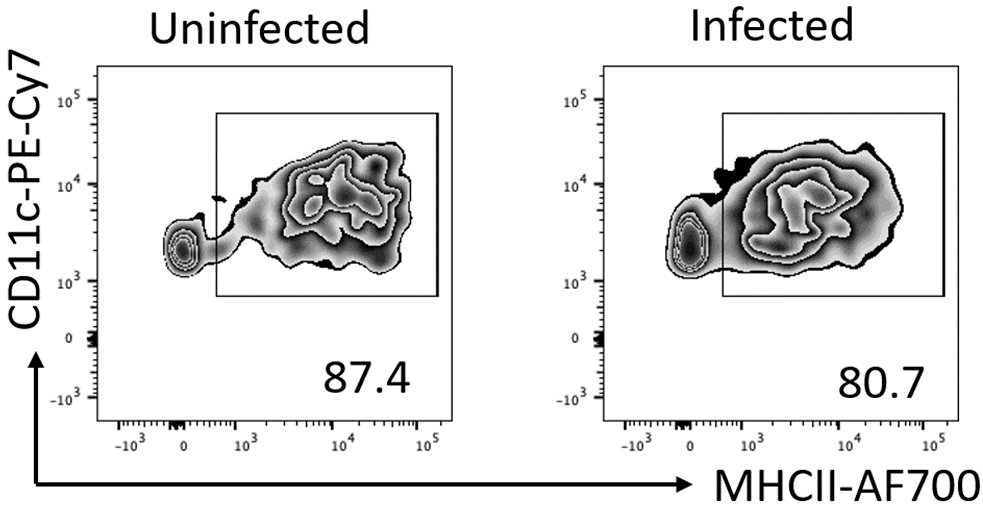
**

**A**


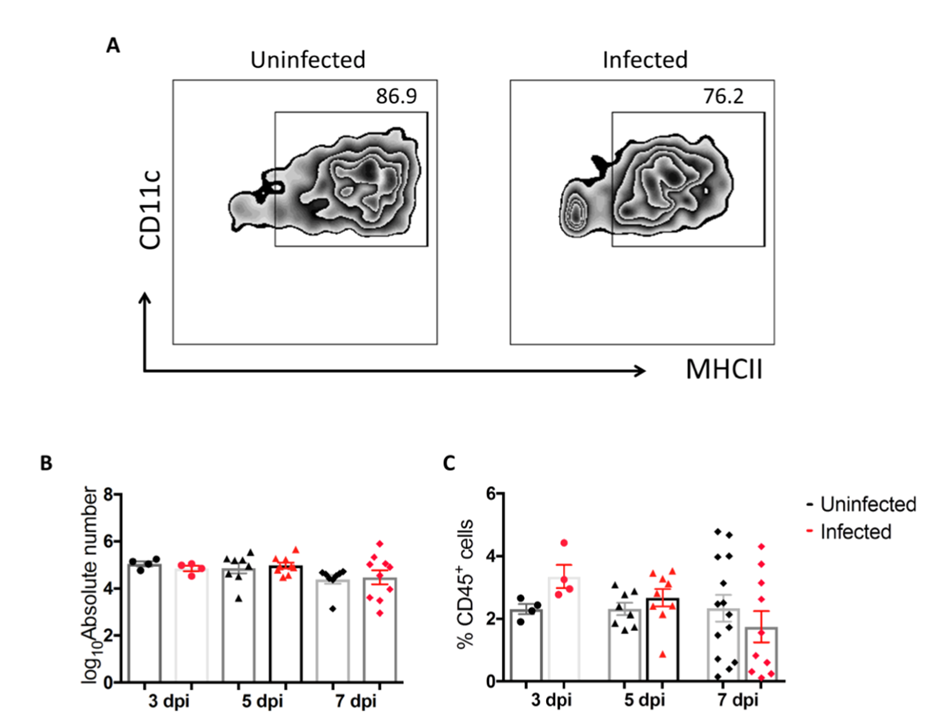


**Figure S4 Effects of SL7207 on tumour dendritic cell content.** Tumour-bearing mice were inoculated with SL7207 or PBS and tumours were harvested at 3, 5 and 7 dpi for flow cytometry analysis of tumour immune cell content. **A.** Representative flow cytometry plots of MHCII+ DCs from infected and uninfected tumours at 5 dpi. Cells were gated on single, live, CD45+, F4/80-, CD11c+. **B.** Quantification of absolute number of CD11c+MHCII+ DCs from infected and uninfected tumours at the indicated time points. **C**. Data shown as percentage CD11c+ MHCII+ DCs of total CD45+ cells from infected and uninfected tumours at the indicated time points. Results displayed are from at least two independent experiments. Results are displayed as the mean ± SEM with each point representing a single animal. Statistical analyses performed using Students t test between infected and uninfected samples at the same time point where p < 0.05*.

**Figure S5**

**
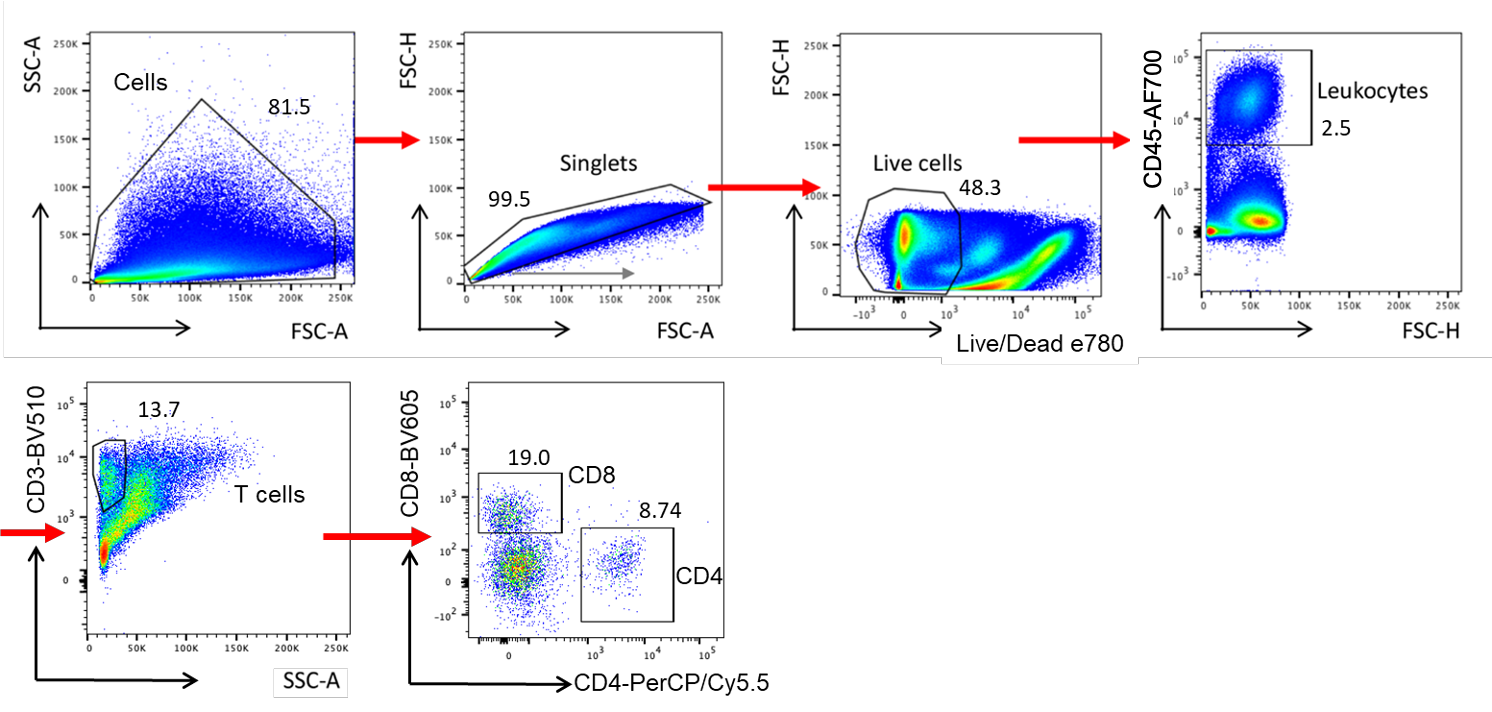
Figure S5 Gating strategies for T cells in the tumour.** Tumour T cells (black and red arrows) were gated as single T cells (black and red arrows) were gated as single cells (side scatter area (SSC-A) versus forward scatter area (FSC-A)), single (FSC-A v forward scatter height (FSC-H), live, CD45+, CD11b+, CD3+, SSClo and CD4+ or CD8+.

**Figure S6**


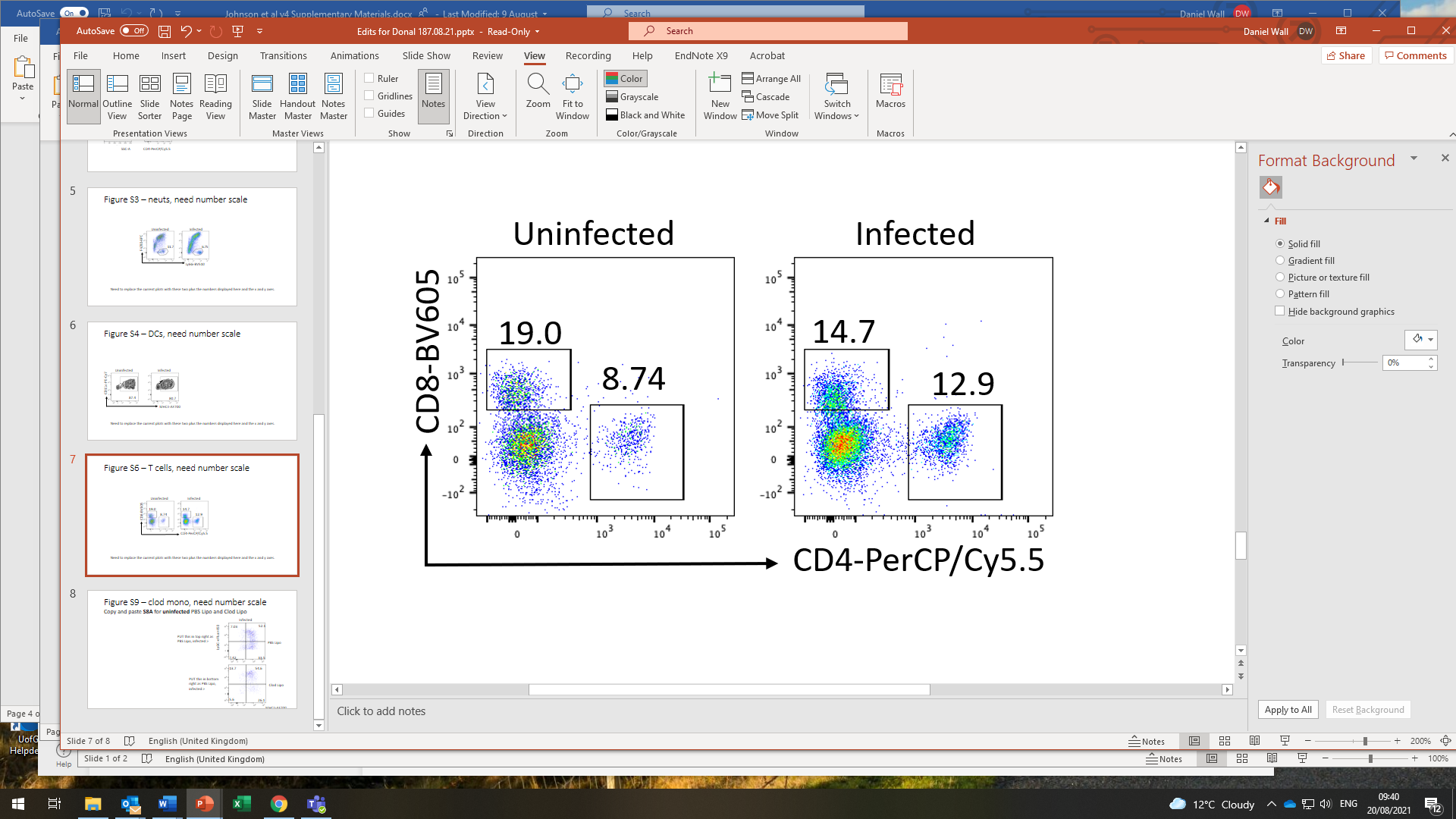


**A**

**
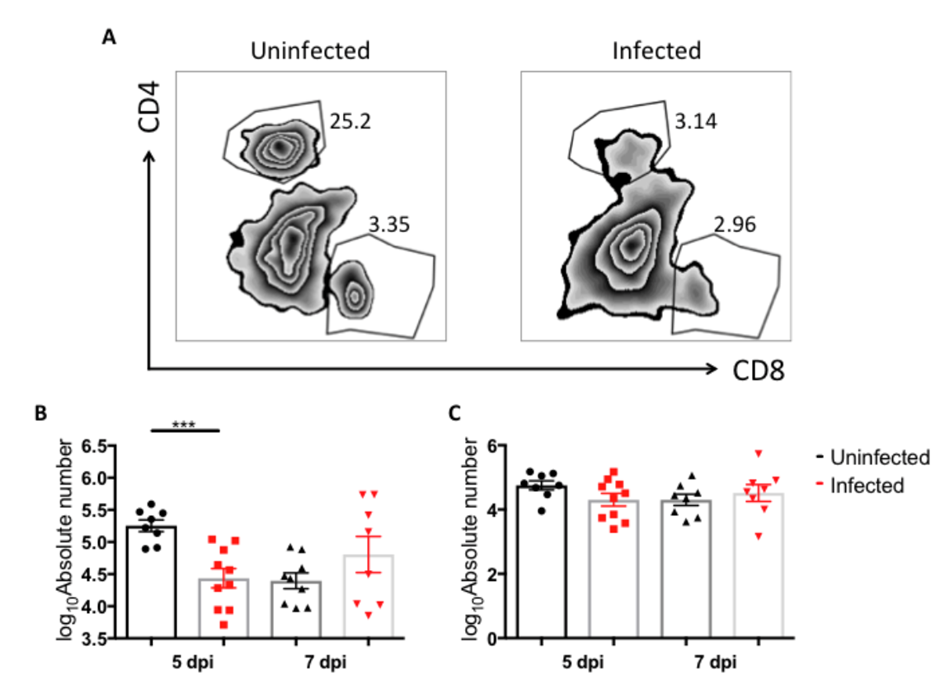
**

**Figure S6 Effects of SL7207 on tumour T cell content.** Tumour-bearing mice were inoculated with SL7207 or PBS and tumours were harvested at 5 and 7 dpi for flow cytometry analysis of tumour T cell content. **A.** Representative flow cytometry plots showing CD4+ and CD8+ T cells from infected and uninfected tumours. Cells were gated as CD45+, CD3+, SSClo. **B.** Quantification of absolute number of CD4+ T cells from infected and uninfected tumours at the indicated time points. **C**. Quantification of absolute number of CD8+ T cells from infected and uninfected tumours at the indicated time points. Results displayed are from at least two independent experiments. Results are displayed as the mean ± SEM with each point representing a single animal. Statistical analyses performed using Students t test between infected and uninfected samples at the same time point where p < 0.05*, p < 0.01**, p < 0.001***.

**Figure S7**

**
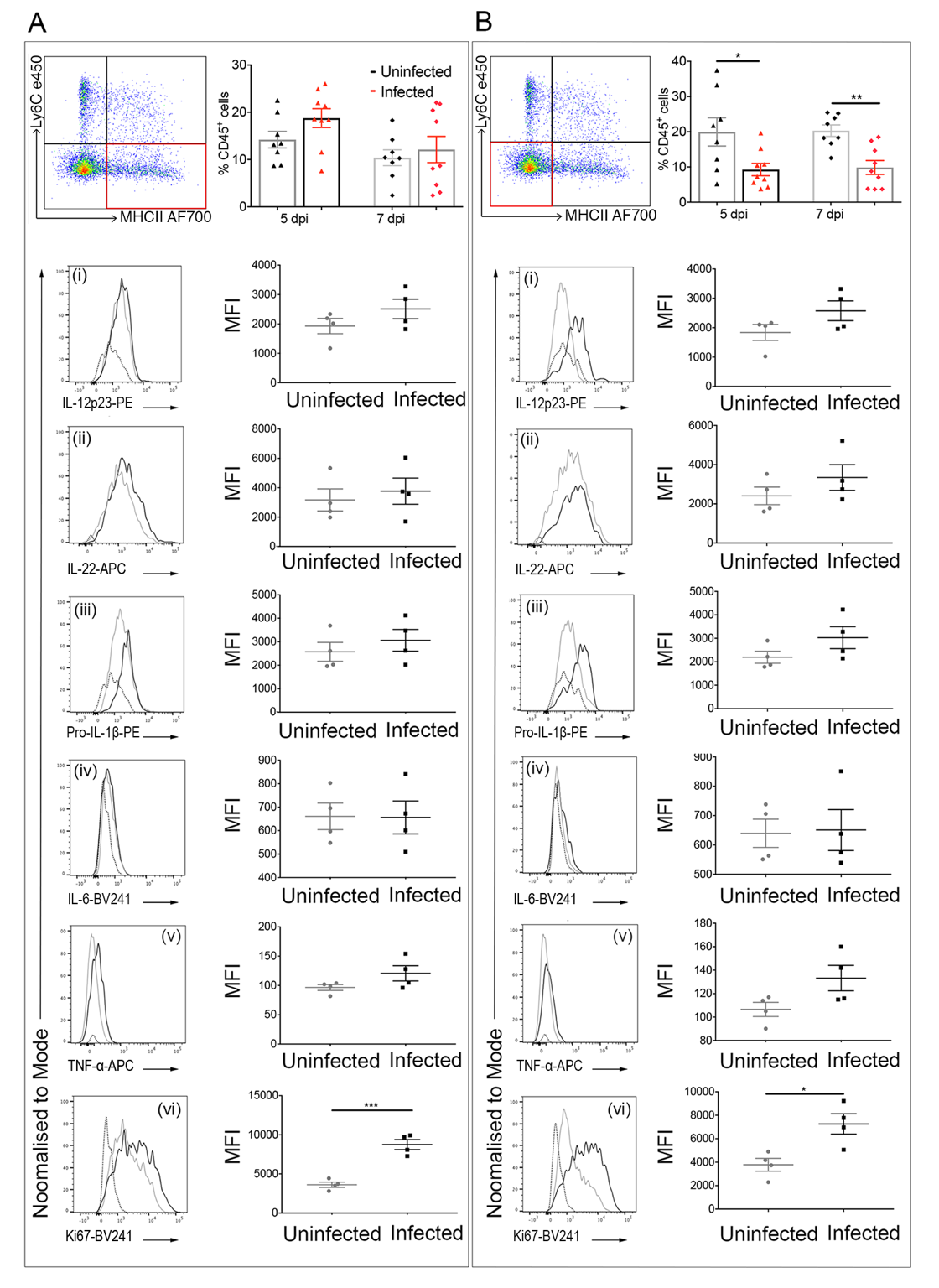
**

**Figure S7 Changes in inflammatory profile of mature TAMs following infection.** These data are related to Fig. 3. (**A**) Depiction of relevant cell population at 7 dpi (Ly6C^-^MHCII^+^ macrophages; left panel) with number of cells as a percentage of CD45^+^ cells (right panel; n= at least 8) and the relative expression of (**i**) IL-12p23, (**ii**) IL-22, (**iii**) pro-IL-1β, (**iv**) IL-6, (**v**) TNF-α and (**vi**) Ki67 in this population with a representative plot from each sample group: isotype control (broken grey line), uninfected (light grey line) and infected (dark grey line). (**B**) Depiction of relevant cell population at 7 dpi (Ly6C^-^MHCII^-^ macrophages; left panel) with number of cells as a percentage of CD45^+^ cells (right panel; n= at least 8) and the relative expression of (**i**) IL-12p23, (**ii**) IL-22, (**iii**) pro-IL-1β, (**iv**) IL-6, (**v**) TNF-α and (**vi**) Ki67 in this population with a representative plot from each sample group: isotype control (broken grey line), uninfected (light grey line) and infected (dark grey line). Samples for cytokine analysis were prepared in Cell Stimulation Cocktail (eBioscience). Cell plots displayed (**A** & **B**) show data representative of two independent experiments; all graphs (**A** & **B**) show results from two independent experimental replicates ± SD; all plots (**Ai-vi** & **Bi-vi**) show quantitative data from one experiment representative of two independent experiments. Samples were analysed using a Student’s t-test where **p*<0.05; ***p*<0.01; ****p*<0.001; and *****p*<0.0001.

**Figure S8**


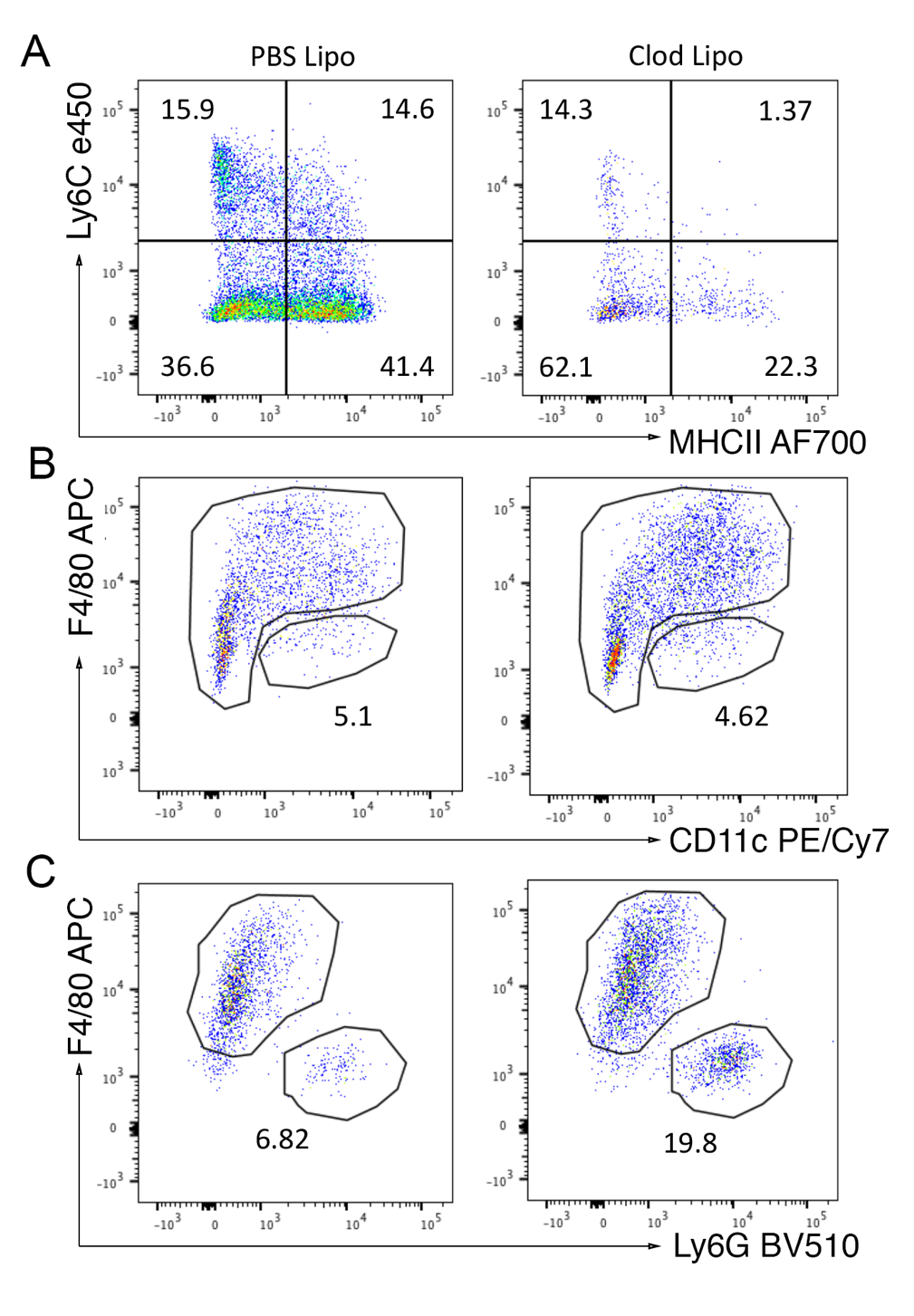


92.2

91.1

88.1

76.6

**Figure S8 Effects of clodronate liposomes on immune cell populations.** (**A**) Flow cytometry analysis of the monocyte/macrophage compartment in tumours following administration of clodronate liposomes or PBS liposomes. (**B**) Flow cytometry analysis of dendritic cells in tumours following administration of clodronate liposomes. (**C**) Flow cytometry analysis of the neutrophils in tumours following administration of clodronate liposomes. Results displayed (**A**, **B** & **C**) are representative of two independent experiments.

**Figure S9**

**
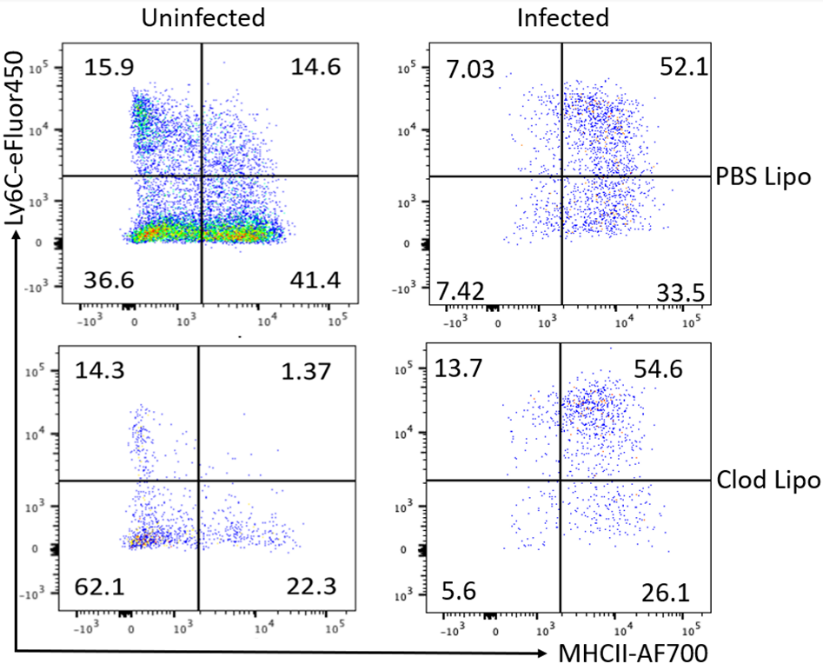
**

**A**


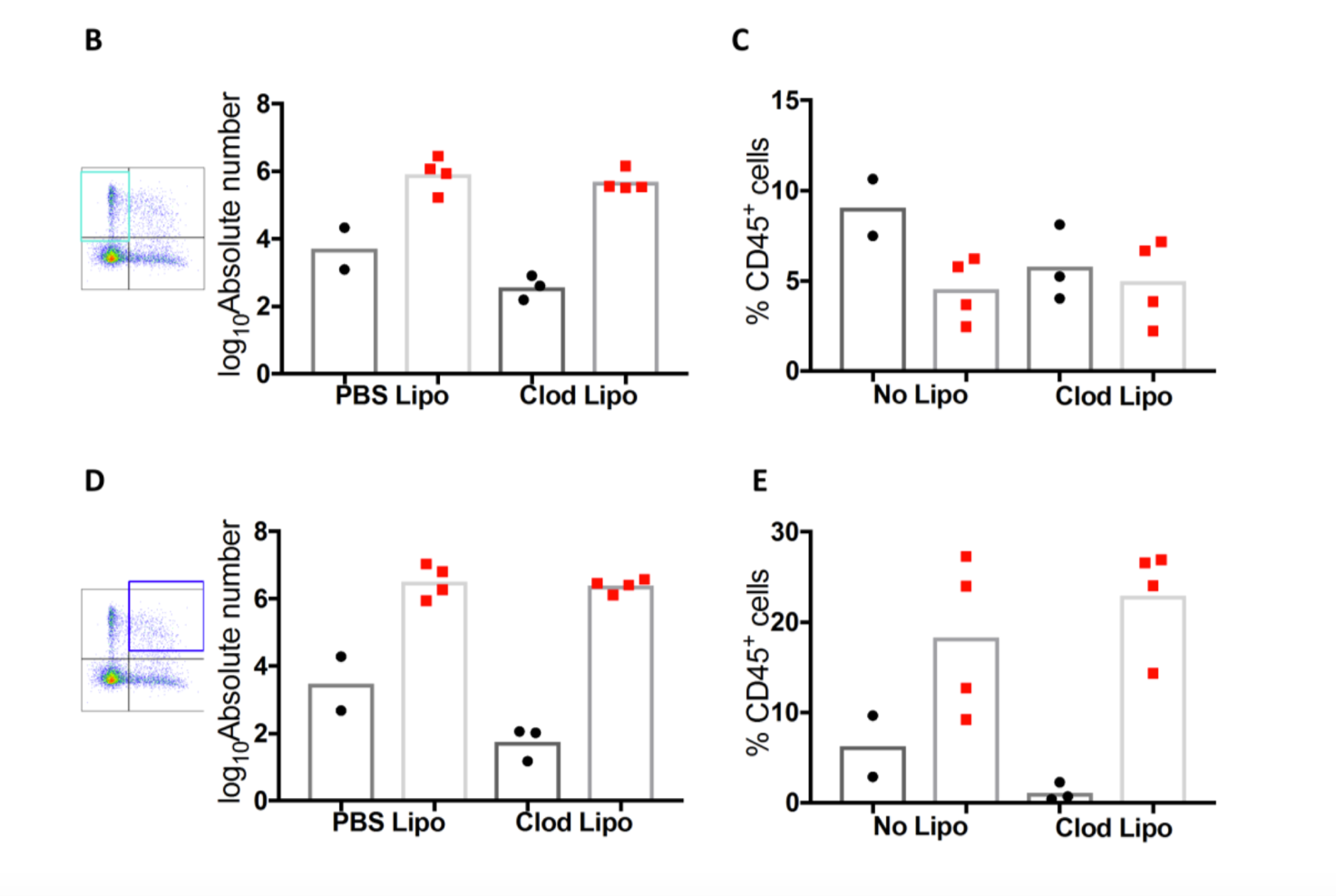


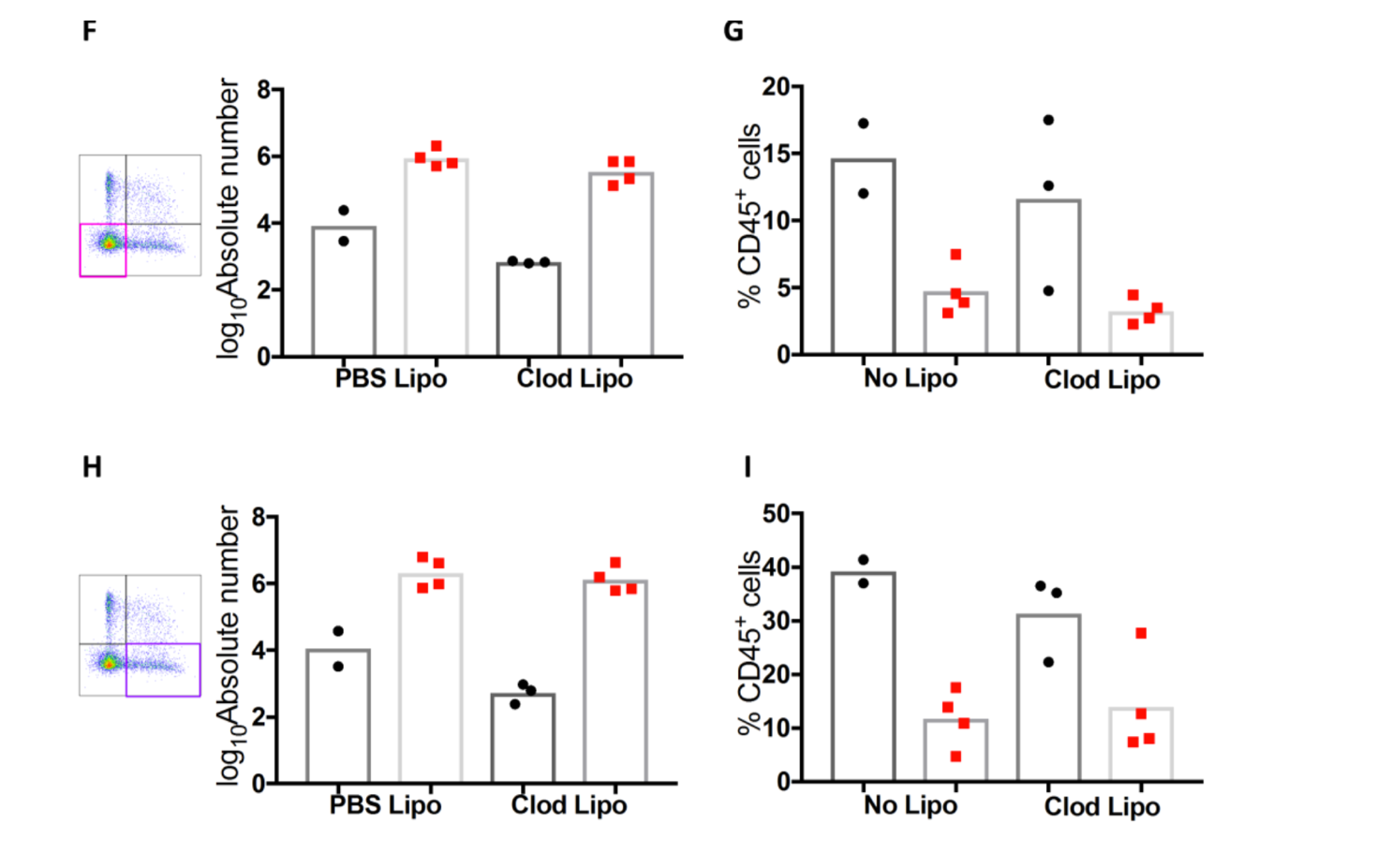


**Figure S9 Effects of SL7207 on the tumour monocyte/macrophage compartment in clodronate liposome treated, SL7207-infected mice.** Tumour-bearing mice, intravenously injected with either PBS Lipo or Clod Lipo were also inoculated with SL7207 or PBS control, and tumours were harvested at 7 dpi for flow cytometry analysis of tumour monocyte/macrophage compartment. Cells were gated on single, live, CD45+, CD11b+, SiglecF-, F4/80+, Ly6G-. **A.** Representative flow cytometry plots of tumour monocyte/macrophage compartment from PBS Lipo and Clod Lipo-treated mice, infected and uninfected. **B.** Quantification of absolute number of Ly6C+MHCII- monocytes in PBS Lipo and Clod Lipo, infected (red) and uninfected (black). **C.** Data shown as percentage Ly6C+MHCII- monocytes of total CD45+ cells in PBS Lipo and Clod Lipo, infected (red) and uninfected (black). **D.** Quantification of absolute number of Ly6C+MHCII+ monocytes in PBS Lipo and Clod Lipo, infected (red) and uninfected (black). **E.** Data shown as percentage Ly6C+MHCII+ monocytes of total CD45+ cells in PBS Lipo and Clod Lipo, infected (red) and uninfected (black). **F.** Quantification of absolute number MHCII- TAMs in PBS Lipo and Clod Lipo, infected (red) and uninfected (black). **G.** Data shown as percentage MHCII- TAMs of total CD45+ cells in PBS Lipo and Clod Lipo, infected (red) and uninfected (black). **H.** Quantification of absolute number MHCII+ TAMs in PBS Lipo and Clod Lipo, infected (red) and uninfected (black). **I.** Data shown as percentage MHCII+ TAMs of total CD45+ cells in PBS Lipo and Clod Lipo, infected (red) and uninfected (black). Statistical analysis performed using Student’s t test between infected PBS Lipo and infected Clod Lipo samples where p < 0.05*.

**Figure S10**

**Figure S10**  **Effect of SL7207 on the tumour dendritic cell population in clodronate liposome treated, SL7207-infected mice.** Tumour-bearing mice, intravenously injected with either PBS Lipo or Clod Lipo were also inoculated with SL7207 or PBS control, and tumours were harvested at 7 dpi for flow cytometry analysis of tumour monocyte/macrophage compartment. Cells were gated on single, live, CD45^+^, F4/80^-^, CD11c^+^. Data shown as percentage dendritic cells of total CD45^+^ cells in PBS Lipo and Clod Lipo, infected (orange/purple squares) and uninfected (green/blue circles). Results displayed are from at least two independent experiments with each point representing a single animal. Statistical analysis performed using Student’s t test between infected PBS Lipo and infected Clod Lipo samples where p < 0.05*.

**Figure S11**

**Figure S11**  **Effect of SL7207 on the tumour neutrophil population in clodronate liposome treated, SL7207-infected mice.** Tumour-bearing mice, intravenously injected with either PBS Lipo or Clod Lipo were also inoculated with SL7207 or PBS control, and tumours were harvested at 7 dpi for flow cytometry analysis of tumour monocyte/macrophage compartment. Cells were gated on single, live, CD45^+^, F4/80^-^, Ly6G^+^. Data shown as percentage dendritic cells of total CD45^+^ cells in PBS Lipo and Clod Lipo, infected (orange/purple squares) and uninfected (green/blue circles). Results displayed are from at least two independent experiments with each point representing a single animal. Statistical analysis performed using Student’s t test between infected PBS Lipo and infected Clod Lipo samples where p < 0.05*.

**Figure S12**


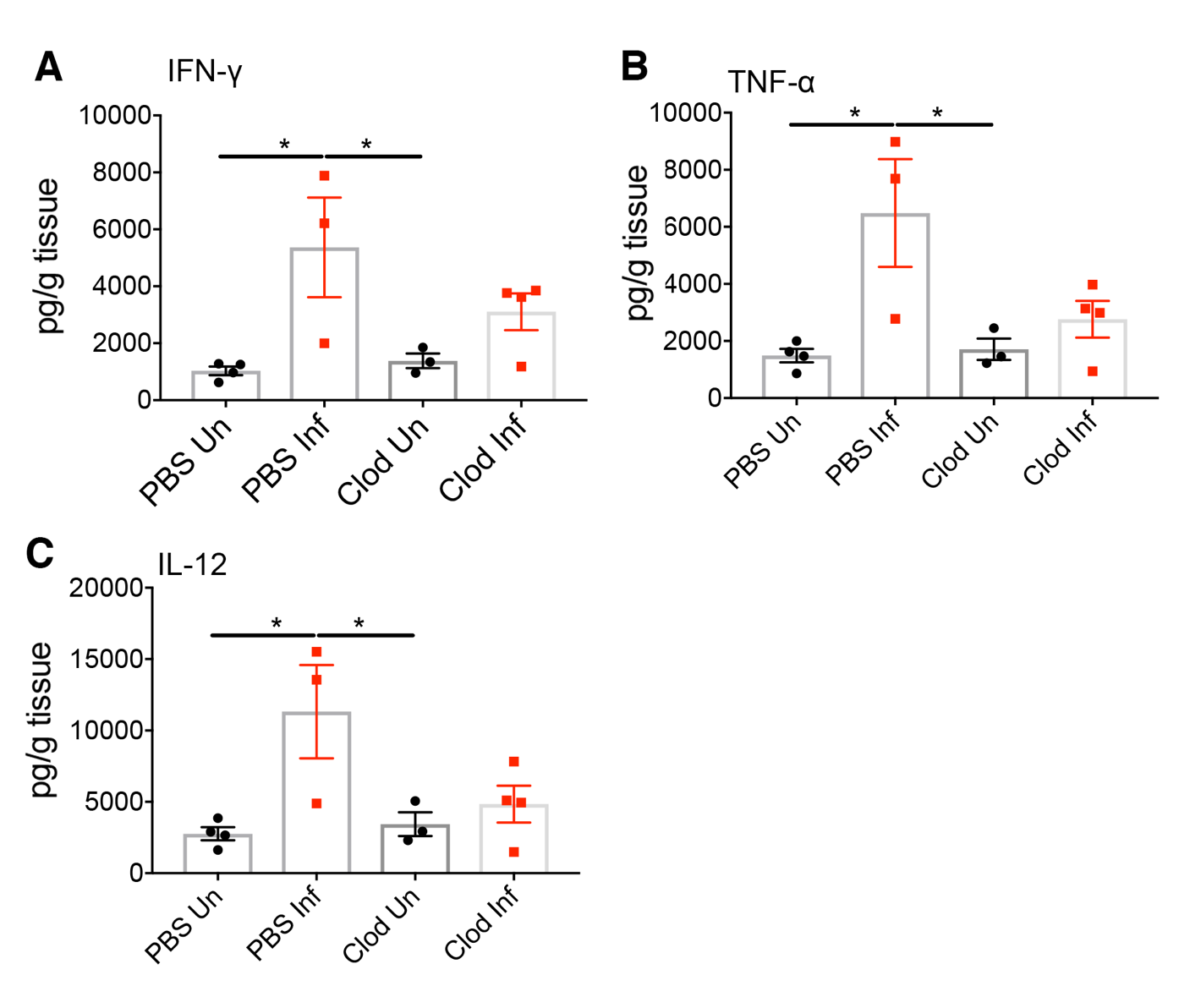
**Figure S12** **Clodronate liposome (Clod lipo) administration protects tumours from SL7207-mediated growth inhibition.** B16F10 melanoma tumours were allowed to develop in C57BL/6 mice (n= at least 3) with serial measurements and were subjected to PBS Lipo administration or Clod Lipo administration (blue arrows) with or without SL7207 infection (purple arrow). (Un = uninfected, In = infected) (**A**) ELISA analysis of tumour lysates at 7 dpi for IFN-γ and (**B**) TNF-α. (**C**) ELISA analysis of tumour lysates at 7 dpi for IL-12p23. Samples for cytokine analysis were stimulated with Cell Stimulation Cocktail (eBioscience). Results displayed are from one experimental replicate (n= at least 3). Results are displayed as the mean ± SD with each point representing a single animal. Samples were analysed a one-way ANOVA where **p*<0.05.
